# Growth hormone (GH) replacement in hypopituitary adults with GH deficiency evaluated by a utility-weighted quality of life index: a precursor to cost–utility analysis

**DOI:** 10.1111/j.1365-2265.2007.03010.x

**Published:** 2008-01

**Authors:** Maria Kołtowska-Häggström, Paul Kind, John P Monson, Björn Jonsson

**Affiliations:** * KIMS Medical Outcomes, Pfizer Endocrine Care Sollentuna, Sweden; † Department of Pharmacy, Uppsala University Uppsala, Sweden; ‡ Outcomes Research Group, Centre for Health Economics, University of York York, UK; § Department of Endocrinology, St Bartholomew's Hospital, Queen Mary University of London London, UK; ¶ Department of Women's and Children's Health, Uppsala University Uppsala, Sweden

## Abstract

**Objectives:**

To examine quality of life (QoL) measured by a utility-weighted index in GH-deficient adults on GH replacement and analyse the impact of demographic and clinical characteristics on changes in utilities during treatment.

**Design:**

Utilities for items in the QoL-Assessment of Growth Hormone Deficiency in Adults (QoL-AGHDA_utility_) were estimated based on data obtained from the general population in England and Wales (E&W). These estimates were used to calculate QoL changes in GH-treated patients and compare these with normative population values.

**Patients:**

A total of 894 KIMS patients (53% women) from E&W were followed for 1 to 6 years.

**Measurements:**

QoL-AGHDA_utility_ at baseline and at the last reported visit, total QoL-AGHDA_utility_ gain and QoL-AGHDA_utility_ gain per year of follow-up.

**Results:**

QoL-AGHDA_utility_ in patients before GH treatment differed from the expected population values [0·67 (SD 0·174) *vs.* 0·85 (SD 0·038), *P* < 0·0001], constituting a mean deficit of –0·19 (SD 0·168). There was a difference in the mean QoL-AGHDA_utility_ deficit for men [–0·16 (SD 0·170)] and women [–0·21 (SD 0·162)] (*P* < 0·001). The main improvement occurred during the first year of treatment [reduction of a deficit to –0·07 (SD 0·163) (*P* < 0·001) in the total cohort]; however, patients’ utilities remained lower than those recorded for the general population during subsequent follow-up (*P* < 0·001). Despite an observed impact of age, primary aetiology, disease onset and comorbidities on QoL-AGHDA_utility_, all patients showed a similar beneficial response to treatment.

**Conclusions:**

QoL-AGHDA_utility_ efficiently monitors treatment effects in patients with GHD. The study confirmed the QoL-AGHDA_utility_ deficit before treatment and a similar QoL-AGHDA_utility_ gain observed after commencement of GH replacement in all patients.

## Introduction

**Fig. 1:**
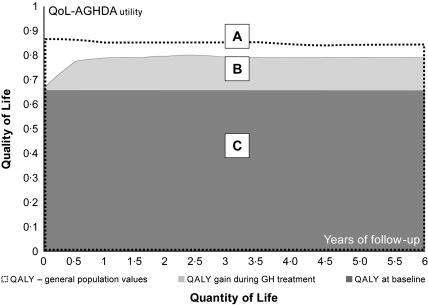
The area under the curve represents QALYs measured by QoL-AGHDA_utility_ during 6 years of treatment, where A depicts general population values, B gain during GH replacement, and C values for patients with GHD without treatment. The value 1 on the *y*-axis stands for full health, and 0 for death.

Quality of life (QoL) has emerged as an important construct that has found numerous applications across health care-related fields, ranging from randomized controlled trials, as well as pharmacoeconomic evaluation, through to daily clinical practice. Each of these applications imposes different requirements on the QoL measures. Pharmacoeconomic evaluation often requires that health status is expressed as a single summary score (a health status index) that is capable of identifying and quantifying differences across diseases as well as aggregate changes in health status over time in patients.^1^ By contrast, clinical applications usually require a measure that captures specific changes within a certain disease, in patient populations (in clinical trials) and in individual patients (in daily clinical practice).^2^ Economic applications impose further requirements when the effects of health care are assessed by cost–utility analysis. Here it is expected that such effects are expressed in terms of quality-adjusted life years (QALYs). This unit of measure combines information on length of life (quantity) and quality of life, where the latter is measured on a scale that has values of 1 and 0, respectively, for full health and death (Fig. 1). The unit QALY is therefore defined as 1 year of life with full health. When a QoL index is used to calculate QALY benefits, health economists also require that the value of health should be estimated in terms of utility weights using preference measurement techniques such as Time Trade-Off (TTO) or Standard Gamble (SG). In summary, economists make different requirements of QoL measurement compared with clinicians, principally as a result of their different information needs.

As impairment in QoL is a key clinical feature of growth hormone deficiency (GHD) in adults, 3, 4 these theoretical aspects of QoL assessment are becoming a matter of significance for all stakeholders. Disease-orientated measures such as the QoL-Assessment of GHD in Adults (QoL-AGHDA)^5^ and the Questions on Life Satisfaction Hypopituitarism Module (QLS-H)^6^ have been developed for use by clinicians, while health economists continue to rely on preference-based approaches to measure QoL in this group of patients.^7^, ^8^

The interrelationship between preference-based QoL assessments and results derived by disease-sensitive instruments is of interest to researchers, mainly because of its numerous practical implications.^9^–^11^ However, in endocrinology such research seems to have been relatively neglected and as far as we are aware has not been undertaken in the field of adult GHD. Our study aimed to bridge the gap between clinicians and health economists by addressing the issue of consistency between QoL measures as used in clinical practice and QALYs. We also examined the QoL deficit, measured by a utility-weighted index (utility) in GH-treated hypopituitary adults with GHD in relation to the general population, and the impact of demographic and clinical characteristics on the change in utilities during GH treatment.

## Methods and subjects

### Study design

The study consisted of two parts, the first of which estimated utilities for the QoL-AGHDA based on data obtained from a survey of the general population in England and Wales (E&W). The second part used utility-weighted QoL-AGHDA data to calculate QoL changes in patients during treatment in relation to normative population values, also examining the impact of demographic and patients’ clinical characteristics.

### General population – deriving utilities

EQ-5D, 12 a generic measure of QoL developed by the EuroQoL group, defines a total of 243 health states for each of which there is a corresponding score based on values obtained from the UK general population, using TTO methods.^13^ Based on these data a set of utilities for all health states described by the EQ-5D has been estimated.

The QoL-AGHDA consists of 25 items that evoke yes/no answers to specific problems. A total score is produced by summing across all items; a high QoL-AGHDA score denotes a poor QoL.

A questionnaire package containing EQ-5D and QoL-AGHDA was sent out to 1190 individuals from a general population in E&W^14^ (response rate 84%). For the purpose of this study, responses from 921 individuals (56% women) who returned complete EQ-5D and QoL-AGHDA questionnaires were included. The mean age (years) of the participants was 53·8 (SD 14·28): 56·3 (SD 14·05) for men and 51·7 (SD 14·16) for women.

A regression model was used to estimate utility weights for QoL-AGHDA items. The TTO-weighted EQ-5D_index_ was used as the dependent variable and QoL-AGHDA item responses were entered as independent dummy variables together with age as a covariate.

(1) $${\text{QoL-AGHDA}}_{\text{utility}}\to {\text{ED-5D}}_{\text{index}}=b_{0}+\left(c\times \text{age}\right)+\Sigma b_{i}\times x_{i}+e_{i}$$

where *x_i_* (*i* = 1–25) correspond to the 25 dichotomous items (coded as 0 = no or 1 = yes) that are summed to form the QoL-AGHDA score, *b_i_* are the regression coefficient estimates, and *e_i_* correspond to error terms.

The model demonstrated an adjusted *R*^2^ of 0·42. Each regression coefficient, *b_i_*, represents the utility weight for the corresponding QoL-AGHDA item and when aggregated across all 25 items, this yields an estimate of the utility-weighted QoL-AGHDA, referred to here as QoL-AGHDA_utility_.

### Adult hypopituitary patients

The patients were retrieved from the KIMS (Pfizer International Metabolic Database).^15^ Data were collected on specially designed case report forms and monitored by study monitors. Each KIMS centre obtained approval from its local ethics committee and patients gave informed consent, either verbally or in writing, depending on the local legal requirements.

Model (1) was used to transform patients’ QoL-AGHDA scores into QoL-AGHDA_utility_. Additionally, QoL-AGHDA_utility_ was correlated against Psychological General Well-Being^16^ (PGWB) scores. The PGWB is a 22-item questionnaire with higher values indicating more satisfactory feelings. The total score ranges from 22 to 132 (with higher scores representing better psychological well-being).

### Calculating QALYs

Patients had been followed in the KIMS database for a varying number of years. The total QoL-AGHDA_utility_ for each patient was calculated using the trapezoid formula as follows (Fig. 1).

(2) $$\Sigma (u_{i-1}-2u_{0}+u_{i})/2\;\;\;\left(i=1,t\right)$$

where *t* = total duration of patient follow-up in KIMS, and *u_i_* = QoL-AGHDA_utility_ at year *i*.

The average change in QALYs over the entire time period was also computed as gain per year. Missing observations between years were substituted using the last observation carried forward (LOCF) technique. The calculation was performed conservatively, assuming that an untreated patient would stay in the same QoL stage as baseline observation. The patient QALY deficit was calculated as the difference between the QoL-AGHDA_utility_ observed in patients and the corresponding value computed for age/gender-matched individuals in the general population sample.

A high QoL-AGHDA_utility_ score denotes better QoL assessment, which is contrary to the interpretation of a QoL-AGHDA raw score, where a high value indicates poor QoL.

### Patients subgroups

Finally, QoL-AGHDA_utility_ at baseline and following GH treatment was evaluated with respect to age, gender, primary aetiology, onset of pituitary disease (childhood *vs.* adulthood), extent of hypopituitarism and medical history.

### Statistics

Parametric statistics were applied when analysing differences between subgroups.

Descriptive statistics are given as mean (SD). One-sample and independent samples *t*-tests and one-way analysis of variance (ANOVA) were used, as well as Pearson correlation analysis. Multiple forward regression analysis was also applied to control for the influence of age and gender on QALYs. A *P*-value less than 0·05 was considered statistically significant.

IGF-I concentrations are described as SD scores. SD scores are calculated as [observed serum IGF-I level – population mean serum IGF-I level (standardized for age and gender)]/population standardized standard deviation.

## Results

The mean observed EQ-5D_index_ in the general population [0·83 (SD 0·214) in men and 0·81 (SD 0·228) in women] reflected closely the estimated QoL-AGHDA_utility_[0·83 (SD 0·127) in men and 0·83 (SD 0·141) in women].

### Patient characteristics

The patient cohort consisted of 894 participants (53% women) from E&W who were followed up for 1 to 6 years. Mean observation time was 3·4 (1·74) years. All patients had GHD confirmed by relevant stimulation tests and were not treated with GH for a minimum of 6 months prior to entry.

The mean age of the patients was 40 (SD 16·5) years at diagnosis of GHD, and 45 (SD 14·3) years at entry into KIMS. Men were slightly older than women at both time points: at diagnosis men were aged 41 (SD 17·1) and were women 40 (SD 15·9), and at entry into KIMS men were 45 (SD 14·7) and women were 44 (SD 13·9).

Detailed information about primary aetiology, according to the KIMS classification list, 17 is presented in Table 1. Almost 40% of patients received surgery or irradiation for treatment of their primary disease. Most of the patients developed their disease during adulthood; only 21·6% had childhood-onset (CO) GHD. Isolated GHD was present in 13·2% of patients, GHD plus one or two other pituitary deficits was present in 16·7% and 17·7% of patients, respectively. Close to 35% of patients were deficient in GH and three other pituitary hormones, whereas 17·4% had panhypopituitarism.

**Table 1:** QoL-AGHDA_utility_ scores (absolute and change) at baseline and at the last reported visit by primary aetiology for hypopituitarism according to the KIMS Classification List. Data shown as mean (SD)

	N	%	Baseline visit	Last reported	Total gain	Gain/year
Nonfunctioning pituitary adenoma	201	22·5	0·64 (0·169)	0·76 (0·172)	0·36 (0·537)	0·10 (0·121)
Secreting pituitary adenoma	311	34·8	0·64 (0·165)	0·76 (0·166)	0·36 (0·592)	0·09 (0·124)
Other sellar	64	7·2	0·68 (0·182)	0·78 (0·183)	0·30 (0·498)	0·09 (0·124)
Craniopharyngioma	91	10·2	0·71 (0·172)	0·80 (0·172)	0·31 (0·602)	0·07 (0·123)
Extracellar tumour	55	6·2	0·69 (0·163)	0·76 (0·194)	0·18 (0·379)	0·06 (0·101)
Idiopathic GHD	58	6·5	0·75 (0·171)	0·82 (0·165)	0·28 (0·563)	0·07 (0·119)
Treatment for malignancy outside the cranium	20	2·2	0·72 (0·21)	0·82 (0·174)	0·08 (0·654)	0·07 (0·17)
Other causes of acquired GHD	94	10·5	0·68 (0·179)	0·80 (0·154)	0·24 (0·422)	0·07 (0·118)
Total	894	100·0	0·67 (0·174)	0·77 (0·171)	0·32 (0·549)	0·08 (0·122)

Gonadotrophin deficiency was present in 68·9% of patients, TSH in 66·9%, ACTH in 65·2% and antidiuretic hormone (ADH) in 25·3%. All pituitary hormone deficits were routinely replaced.

Fractures were reported in 40%, hypertension in 19%, heart problems in 12%, asthma and/or allergy in 12%, arthrosis in 10% and diabetes mellitus in 6% of the KIMS patients. Overall, 57% of KIMS patients reported one concomitant disease and 20% more than one.

The mean maintenance GH dose (defined as the dose at the 1-year visit) of 0·44 (SD 0·220) mg/day in female patients resulted in a change in serum IGF-I from a mean SD score of –2·26 (SD 1·782) to 0·25 (SD 1·496) whereas the lower GH dose [0·37 (0·185) mg/day] in male patients increased serum IGF-I from a mean SD score of –1·40 (SD 1·915) to 0·52 (SD 1·507).

### QoL-AGHDA_utility_

The details of raw QoL-AGHDA scores in patients are presented in Table 2.

**Table 2:** QoL-AGHDA and QoL-AGHDA_utility_ scores (absolute and change) at baseline and at last reported visit by gender and for the total cohort

		QoL-AGHDA score*				QoL-AGHDA_utility_ score†			
			
		Baseline visit	Last reported	Total decrease	Decrease/year	Baseline visit	Last reported	Total gain	Gain/year
Men (*n* = 423)	Mean (SD)	13·9 (6·58)	8·7 (6·91)	–5·2 (6·44)	–2·4 (4·11)	0·70 (0·174)	0·79 (0·165)	0·25 (0·473)	0·07 (0·113)
	Median	15·0	8·0	–5·0	–1·3	0·71	0·86	0·16	0·06
Women (*n* = 472)	Mean (SD)	15·9 (6·10)	9·45 (6·99)	–6·4 (7·02)	–2·7 (3·89)	0·63 (0·166)	0·76 (0·174)	0·38 (0·602)	0·10 (0·129)
	Median	16·5	9·0	–6·0	–1·7	0·63	0·81	0·29	0·11
	*P*_B_	< 0·001	NS	< 0·002	NS	< 0·001	< 0·003	< 0·001	< 0·001
Total (*n* = 895)	Mean (SD)	14·9 (6·40)	9·1 (6·96)	–5·8 (6·78)	–2·6 (4·00)	0·67 (0·172)	0·77 (0·171)	0·32 (0·549)	0·08 (0·122)
	Median	16·0	8·0	–5·0	–1·5	0·66	0·83	0·22	0·09

All changes within groups (paired *t*-test) were significant, *P* < 0·001.

*P*_B_, significance of differences between groups (independent *t*-test).

* A low QoL-AGHDA score indicates good QoL, meaning that a decrease in score denotes improvement in QoL.

† A high QoL-AGHDA_utility_ score indicates good QoL, meaning that an increase in score denotes improvement in QoL.

The mean QoL-AGHDA_utility_ score at baseline was 0·67 (SD 0·172). Women scored significantly lower than men [0·63 (SD 0·166) *vs*. 0·70 (SD 0·174), *P* < 0·001], indicating a worse QoL. The total observed QALY gain was higher for women than men [0·38 (SD 0·602) *vs.* 0·25 (SD 0·473), *P* < 0·001], as was the mean change in QoL-AGHDA_utility_ per year [0·10 (SD 0·129) *vs*. 0·07 (SD 0·113), *P* < 0·001]. All within-group changes were significant (*P* < 0·001), as shown in Table 2.

There were significant (*P* < 0·0001) positive correlations between PGWB scores at baseline, last observation and change in PGWB score and corresponding measures of QoL-AGHDA_utility_ (*r* = 0·68, *r* = 0·68 and *r* = 0·42, respectively). These highly significant correlations indicate consistency between both measures; that is, if psychological well-being as measured by PGWB improves, QoL-AGHDA_utility_ also shows improvement and vice versa.

### Comparison of QoL-AGHDA_utility_ in patients and the general population

QoL measured by QoL-AGHDA_utility_ in patients before commencement of GH treatment differed significantly from the expected values calculated from the sample of the general population [0·67 (SD 0·174) *vs.* 0·85 (SD 0·038), *P* < 0·0001], constituting a mean deficit of –0·19 (SD 0·168). There was also a significant difference in the mean QoL-AGHDA_utility_ deficit for men [–0·16 (SD 0·170)] and women [–0·21 (SD 0·162)] (*P* < 0·001). The main improvement occurred during the first year of observation when the QoL-AGHDA_utility_ deficit was reduced to –0·07 (SD 0·163) (*P* < 0·001) in the total cohort and to –0·07 (SD 0·160) (*P* < 0·001) in men and –0·08 (SD 0·170) (*P* < 0·001) in women. The difference between genders disappeared after the first year of GH treatment. The same was true for the deficit at the last reported visit: men –0·07 (SD 0·160) and women –0·08 (SD 0·170). Despite a dramatic improvement during the first year of observation that was maintained during the whole follow-up period, patients’ QoL-AGHDA_utility_ remained significantly different (*P* < 0·001) from those reported by the general population (line A in Fig. 1).

### Patient subgroups

In the last step of this study, QoL-AGHDA_utility_ at baseline and response to GH treatment were evaluated with respect to demographic and clinical characteristics.

#### Age

**Fig. 2:**
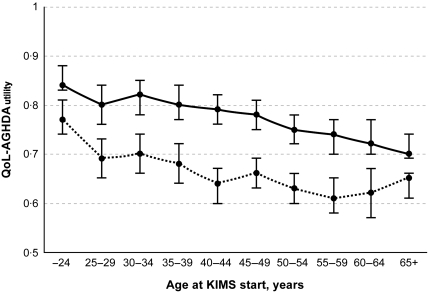
95% confidence intervals for mean QoL-AGHDA_utility_ at baseline (broken line) and at the last reported visit (continuous line) by age group.

QoL-AGHDA_utility_ was negatively correlated with age both at baseline (*r* = ndash 0·23; *P* < 0·0001) and at the latest reported visit (*r* = ndash 0·25; *P* < 0·0001), meaning that QoL-AGHDA_utility_ deteriorated with advancing age (Fig. 2). However, the mean total QoL-AGHDA_utility_ gain and also well as the mean gain per year were similar through all the age groups (data not shown).

#### Primary aetiology

There were differences in QoL-AGHDA_utility_ between aetiology groups at baseline and at the last reported visit. Patients with GHD due to pituitary adenoma, both nonfunctioning and secreting, had the lowest QoL-AGHDA_utility_ at both time points (Table 1). However, the primary cause for GHD had no influence on the response to treatment measured by total QoL-AGHDA_utility_ gain and mean gain per year.

#### Previous treatment

Neither previous surgery nor irradiation had an impact on QoL-AGHDA_utility_ at any time point, and did not influence response to GH (data not shown).

#### Disease onset

**Fig. 3:**
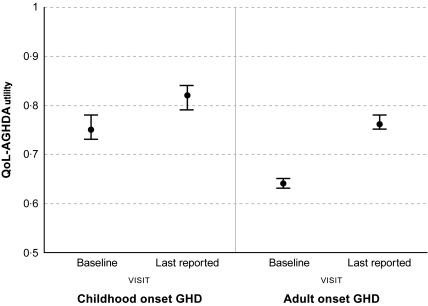
95% confidence intervals for mean QoL-AGHDA_utility_ at baseline and during GH replacement therapy in patients with childhood-onset and adult-onset GHD.

QoL-AGHDA_utility_ scores were higher in patients with CO disease than with adult-onset (AO) both at baseline [0·75 (SD 0·173) *vs.* 0·64 (SD 0·166), *P* < 0·001] and at the last reported visit [0·82 (SD 0·167) *vs.* 0·76 (SD 0·170), *P* < 0·001]. However, patients with CO-GHD gained less than AO patients with regard to the total gain [0·18 (SD 0·488) *vs.* 0·35 (SD 0·559)] and to the mean gain per year [0·05 (SD 0·117) *vs.* 0·09 (SD 0·123)] (Fig. 3).

When controlled for age and gender using multiple regression analysis, patients with CO disease continued to demonstrate significantly higher QoL-AGHDA_utility_ at baseline and responded to a lesser extent to GH treatment than patients with AO (*P* < 0·0001).

#### Extent of hypopituitarism

**Fig. 4:**
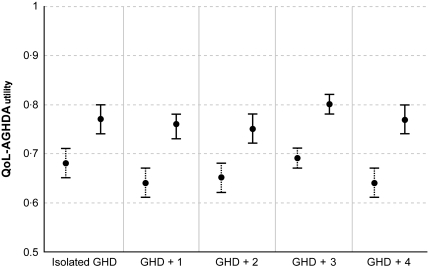
95% confidence intervals for mean QoL-AGHDA_utility_ at baseline (broken line) and during GH replacement therapy (continuous line) in patients by number of pituitary deficits.

The number of additional to GH pituitary hormone deficits showed no significant correlation with any of the QoL-AGHDA_utility_ parameters (Fig. 4). Similarly, patients with isolated GHD demonstrated equivalent levels of deficit in QoL-AGHDA_utility_ at baseline and comparable gain during GH treatment in comparison with patients with multiple pituitary hormone deficiency.

#### Comorbidities

There was a significant impact of reported comorbidities on all QoL parameters. Patients who reported health problems in addition to GHD (*n* = 513) had lower QoL-AGHDA_utility_ mean scores at baseline [0·63 (SD 0·167), *P* < 0·001] and at the last reported visit [0·75 (SD 0·174), *P* < 0·001] compared to patients with no reported comorbidities (*n* = 381) [0·71 (SD 0·172) and 0·81 (SD 0·159), respectively]. At the same time, patients with comorbidities responded better to GH treatment in terms of QoL-AGHDA_utility_[mean 0·36 (SD 0·565) for total gain (*P* < 0·002) and 0·10 (SD 0·124) for gain/year (*P* < 0·004)] compared to patients with no reported comorbidities [0·25 (SD 0·520) and 0·07 (SD 0·119), respectively].

## Discussion

The aim of our study was to investigate clinically assessed QoL in the context of utilities, an outcome used in cost–utility analysis. The first step was to derive utilities directly from a measure that does not *per se* meet pharmacoeconomic requirements but is widely used in clinical practice. We then evaluated patients’ utilities in relation to general population values to assess treatment effects, and finally we analysed different patients’ characteristics to confirm the usefulness of such outcomes in a clinical setting and in detecting any specificity in the patient population with GHD. Additionally, our work was driven by an emerging practical need for a mutual understanding between clinicians and health economists with regard to respective methodology, application and interpretation.

To our knowledge only two previous attempts have been made to derive utilities from the QoL-AGHDA. The first was undertaken by Dixon *et al*., 7 who used a two-step model to link QoL-AGHDA data through the Nottingham Health Profile (NHP) to utilities based on an SF-36 algorithm. However, this method might contain a considerable level of imprecision because of the multiple statistical imputations. In addition, the values originated from patients, whereas utility values used for economic evaluation are usually based on general population values. The second attempt was performed by our group^8^ for a Swedish population. Despite similar methodology, there are two major differences that are worth discussing, namely the choice of values and the model applied.

For estimation of any utilities, it is crucial for the ultimate results that an appropriate set of values for different health states is applied. Two main issues are involved in the choice of values. First, the way they are constructed (SG or TTO), 18, 19 and second, the reference population (patients or general public).^20^ For the former, we decided to use values obtained by the TTO method as it has the requisite basis in theory. For the latter, following the recommendation of the EuroQoL Group, we chose the general population values originating from the same country as our patient cohort (E&W).^21^ In that way we hoped to minimize the impact of possible confounders related to differences in mentality, societal code and culture.

The other methodological issue was the choice of independent variables entered into the regression analysis (the QoL-AGHDA summary score, all individual QoL-AGHDA items or selected items identified in stepwise forward regression analysis). As the final model yielded an adjusted *R*^2^ of 0·42, whereas in a stepwise forward regression analysis and in the model with the QoL-AGHDA summary score the adjusted *R*^2^ assumed the value of 0·40, we decided to choose the model that fitted our data best.

The novelty of our approach is to apply utilities derived from the QoL-AGHDA to the patient population and to evaluate QALY change in a clinical context as a function of treatment response together with patients’ demographic and clinical characteristics.

Cost–utility analysis based on QALY change is the most widely recognized method in pharmacoeconomic evaluation, and QoL-AGHDA was investigated by the National Institute for Health and Clinical Excellence (NICE) as a potential source of outcome data for such an evaluation. Nevertheless, the final conclusion of NICE was that there was a lack of evidence to construct a plausible cost–utility model that would allow cost per QALY to be generated.^22^ Our study, despite its observational nature, which is an obvious limitation, provides a methodology for monitoring QALY changes over the course of GH replacement in comparison with the age- and gender-matched population values. Patients showed a profound QoL deficit before treatment, and significant improvement during follow-up. This pattern is very similar to the pattern of response in QoL measured by QoL-AGHDA^23^ (a dramatic improvement during the first year and a subsequent steady increase during the ensuing years of treatment). The main difference is that the patients’ utilities, contrary to the QoL-AGHDA scores, remained different from the population values during prolonged follow-up. This discrepancy might be related to the nature of both measures, as QoL-AGHDA directly records problems linked to GHD, whereas the utility-weighted index is based on a scoring system that reflects a broader spectrum of health as experienced by the general population. As the duration of follow-up varied from patient to patient, the change in QoL-AGHDA_utility_ was calculated as a total gain per follow-up but also as a gain per year. By doing this, we were able to present results in a more comprehensive way. It is worth noting that the high correlation between PGWB scores and QoL-AGHDA_utility_ constitutes additional evidence for consistency of the methodology.

The last part of our study focused on the impact of demographic and clinical characteristics including gender, age, primary aetiology, onset of the disease, extent of hypopituitarism and comorbidities. Of note, the difference between genders, with women demonstrating lower pretreatment QoL-AGHDA_utility_ (consistent with the results from previously published studies demonstrating that female patients experience worse QoL^24^, ^25^), disappeared during the treatment as the total and mean annual QALY gains were greater in female patients. This observation, in the light of lower overall responsiveness to GH replacement in female patients, 26 further suggests that response in utilities might comprise additional components to those directly related to the symptoms of the disease. However, it should be recognized that gender differences in GH responsiveness are largely eliminated nowadays when GH dose is titrated against serum IGF-I rather than being a fixed quantity based on body size.

As expected, QoL expressed as utilities in younger patients was better (demonstrated by a higher value), which corresponds to many reports on QoL, both for the population and the patients.^27^, ^28^ It is noteworthy that the QoL gain was not affected by age and that older patients benefit equally from GH treatment compared to the younger patients in terms of utilities, supporting previous observations on the QoL response to GH in older patients with hypopituitarism.^29^

Overall, despite some differences at baseline, clinical parameters did not have an impact on response to treatment; all patients presented similar total and annual QoL-AGHDA_utility_ gain. The only exception was patients with CO-GHD and patients with comorbidities. The former responded to GH to a lesser extent. Nevertheless, it should be remembered that patients with CO-GHD were characterized by higher levels of QoL-AGHDA_utility_ at baseline, so it might be speculated that their response was driven by the extent of initial pathology. It has been confirmed that patients with less impairment in QoL at baseline demonstrate minor response.^30^ The same explanation may apply to the observation that patients who reported more comorbidities and thus lower QoL-AGHDA_utility_ at baseline gained more QoL-AGHDA_utility_ during treatment.

Finally, we should highlight the limitations of our study that result from its observational nature and, by definition, lack of randomization. The main warning applies to the potential selection bias that could account for some of this striking variation. In the UK, the major criterion for patient eligibility for GH replacement is impaired QoL and, as KIMS is a database of patients receiving such treatment, levels of QoL and thus QoL-AGHDA_utility_ scores may be worse than in the GH-deficient population at large.

At the same time, it should be borne in mind that causal interpretation of a change observed in any study design other than double-blind, placebo-controlled, and relating it to a treatment, is debatable and should be undertaken with considerable caution. This limitation applies to our study, and we are aware that a clear answer as to whether or not the observed changes were caused by GH treatment can only be given by a controlled study. Nevertheless, in the light of difficulties of conducting such a controlled study in patients with a recognized and approved indication, we believe that the next best option is an observational study using a large number of patients and extensive clinical information. In this way we have attempted to compensate for the lack of placebo-controlled data.

The other limitation of our study relates to the assumption that GH treatment has no differential impact on mortality. QALYs consist of two components, quality (utility) and quantity (duration of life), and both contribute to the final value of the index. The increased mortality rate in hypopituitary patients with untreated GHD has been proven.^31^, ^32^ However, despite promising observations, there is still no final evidence on the beneficial effect of GH replacement on mortality rates. It should be noted that any final QALY estimates should incorporate treatment effects on patients’ survival together with the QoL-AGHDA_utility_ gain presented in this paper. Assuming that GH treatment reverses, at least partly, the increased mortality associated with hypopituitarism, the total QALY gain should account for additional life years.

In conclusion, our study reports a new possibility of translating QoL-AGHDA into utilities. We have shown that this derived QoL-AGHDA_utility_ index, with its main application to cost–utility analysis, efficiently monitors treatment effects in patients with GHD. The study confirmed the QoL-AGHDA_utility_ deficit before treatment and a similar QoL-AGHDA_utility_ gain, despite baseline discrepancies, in all patients observed after commencement of GH replacement.
